# Temperature-Enhanced Exciton Emission from GaAs Cone–Shell Quantum Dots

**DOI:** 10.3390/nano13243121

**Published:** 2023-12-12

**Authors:** Christian Heyn, Leonardo Ranasinghe, Kristian Deneke, Ahmed Alshaikh, Robert H. Blick

**Affiliations:** Center for Hybrid Nanostructures (CHyN), University of Hamburg, Luruper Chaussee 149, 22761 Hamburg, Germanykdeneke@physnet.uni-hamburg.de (K.D.); ahmed.alshaikh@uni-hamburg.de (A.A.); rblick@physnet.uni-hamburg.de (R.H.B.)

**Keywords:** quantum dot, photoluminescence, exciton, biexciton, power dependence, temperature dependence

## Abstract

The temperature-dependent intensities of the exciton (X) and biexciton (XX) peaks from single GaAs cone–shell quantum dots (QDs) are studied with micro photoluminescence (PL) at varied excitation power and QD size. The QDs are fabricated by filling self-assembled nanoholes, which are drilled in an AlGaAs barrier by local droplet etching (LDE) during molecular beam epitaxy (MBE). This method allows the fabrication of strain-free QDs with sizes precisely controlled by the amount of material deposited for hole filling. Starting from the base temperature *T* = 3.2 K of the cryostat, single-dot PL measurements demonstrate a strong enhancement of the exciton emission up to a factor of five with increasing *T*. Both the maximum exciton intensity and the temperature $T_{x,max}$ of the maximum intensity depend on excitation power and dot size. At an elevated excitation power, $T_{x,max}$ becomes larger than 30 K. This allows an operation using an inexpensive and compact Stirling cryocooler. Above $T_{x,max}$, the exciton intensity decreases strongly until it disappears. The experimental data are quantitatively reproduced by a model which considers the competing processes of exciton generation, annihilation, and recombination. Exciton generation in the QDs is achieved by the sum of direct excitation in the dot, plus additional bulk excitons diffusing from the barrier layers into the dot. The thermally driven bulk-exciton diffusion from the barriers causes the temperature enhancement of the exciton emission. Above $T_{x,max}$, the intensity decreases due to exciton annihilation processes. In comparison to the exciton, the biexciton intensity shows only very weak enhancement, which is attributed to more efficient annihilation processes.

## 1. Introduction

Semiconductor quantum dots (QDs) are important building blocks for applications in quantum information technology, in which they act as qubits [1] and deterministic sources of single photons [2] or entangled photon pairs [3,4,5,6]. In this field, the QDs are usually operated at liquid helium temperature to preserve clear excitonic features in the optical spectra. But for device applications, an operation at higher temperatures would be desirable to avoid expensive, large, and maintenance-intensive cooling technologies like liquid-helium cryostats or closed-cycle Gifford–McMahon (GM) systems.

However, with increasing temperature *T*, a broadening of the QD excitonic lines is reported as well as the formation of phonon sidebands [7,8,9,10,11]. Furthermore, a substantial degradation of the intensity of the emission from various types of the QDs at higher temperatures is observed [7,8,12,13,14,15,16,17,18]. Both effects seriously interfere with an application of QDs in optical quantum information technology at elevated temperatures.

This study focuses on GaAs cone–shell QDs (CSQDs) fabricated in a self-assembled fashion via local droplet etching [19] during molecular beam epitaxy (MBE). The self-assembled droplet etching of nanoholes into GaAs surfaces was first demonstrated by Wang et al. [20] and the fabrication of strain-free GaAs QDs by filling of droplet-etched nahoholes in AlGaAs with GaAs by us [21]. This type of QDs demonstrates a high degree of single-photon emission and a very low neutral exciton fine-structure splitting [22] which suggests its suitability for applications in quantum information technology [4].

The present temperature-dependent single-dot photoluminescence (PL) experiments show only a slight exciton peak broadening and only weak phonon sidebands. Even more importantly, the optical data establish a significant temperature enhancement of the exciton intensity with a maximum at a temperature around *T* = 30 K. This allows spectroscopy and possible device applications using economical and compact Stirling cryocoolers. Detailed PL measurements are performed to evaluate the temperature-dependent intensities of the exciton and biexciton peaks at varied excitation power *P* and for QDs with varied size. The peak intensities show a complex interplay between *T*- and *P*-dependence. To ensure the excitation power is sufficient, a rising temperature yields an enhancement of the exciton peak intensity up to a factor of five, followed by the expected reduction. For an interpretation of the data and to identify the underlying mechanisms, a model is introduced which quantitatively reproduces the experimental *T*- and *P*-dependence, including the temperature enhancement.

## 2. Experimental Setup

**Figure 1:**
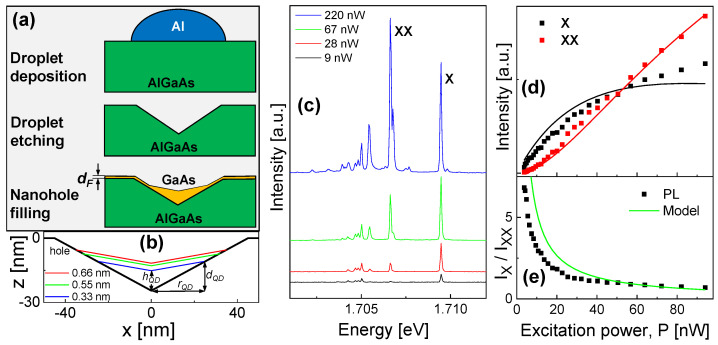
(**a**) Illustration of the fabrication steps for cone–shell quantum dots with the deposition of Al droplets on an AlGaAs substrate, the self-assembled droplet etching of nanoholes, and the deposition of a GaAs layer with thickness $d_{F}$ for nanohole filling. (**b**) Rotational-symmetric shape of a nanohole and of CSQDs with varied $d_{F}$, as calculated according to ref. [23]. (**c**) PL spectra from a single GaAs CSQD with $d_{F}$ = 0.33 nm. The temperature is *T* = 3.2 K and the laser power *P* is varied. The labels indicate the exciton (X) and biexciton (XX) peaks. (**d**) Measured *P*-dependent intensities of the exciton and biexciton peaks (symbols) together with results of model calculations (lines) for a CSQD with $d_{F}$ = 0.33 nm. The intensity represents the peak area of a Lorentzian fit after background subtraction. (**e**) Measured ratio of of the X and XX peak intensities (symbols) as a function of *P* together with model results (line).

The method of local droplet etching during MBE and the usage of this technique for the fabrication of samples with GaAs QDs embedded in an AlGaAs matrix has been described in previous publications [19,21]. In brief, about 30 nm deep cone-like nanoholes with a density of $2\times 10^{7}$ cm${}^{-2}$ are drilled into an AlGaAs substrate (the Al content is 33%) through self-assembled etching with Al droplets. After droplet etching, a GaAs layer with thickness $d_{F}$ is deposited for nanohole filling and generation of the QDs. Here, the thickness $d_{F}$ controls the QD size (Figure 1a) and is varied for the present samples from 0.33 to 0.66 nm. We note that $d_{F}$ is much smaller than the final height of the QDs. The shape and size of droplet etched QDs is discussed in a recent study: ref. [19]. The shapes of CSQDs with the sizes discussed in the present work are calculated according to ref. [23] and shown in Figure 1b. In the present rotational-symmetric approximation, the QD size is characterized by three parameters $h_{QD}$, $d_{QD}$, and $r_{QD}$, as indicated in Figure 1b. The QD size-related parameters for the used filling layer thicknesses are provided in Table 1.

The micro-PL measurements are performed in an optical closed-cycle cryostat (Montana Cryostation S100, Montana Instruments, Bozeman, MT, USA). An integrated temperature controller allows a precise variation of the sample temperature from *T* = 3.2 K up to 350 K. Furthermore, a stack of piezo motors is integrated inside the cryostat for sample movement and QD selection. A green (532 nm) laser is used for the optical exitation of the QDs and the laser power *P* is adjusted by neutral density filters. A power meter measures the laser power, which is corrected for the entrance window of the cryostat. An objective (Olympus LMPLFLN-BD, 100 × 0.8, Edmund Optics, Barrington, NJ, USA) inside the cryostat is used to focus the laser beam and is also used for the collection of the light emitted from the sample. The very low QD density allows the simple selection of individual QDs by the focused laser. A *f* = 500 mm monochromator in combination with an EMCCD camera is used for the analysis of the QD emission.

## 3. Experimental Results

This section addresses measurements of the excitation power and temperature dependence of the exciton and biexcitons peak intensities from different samples with varied QD size. A model of the data is described in Section 4 and a comparison between experimental and model results in Section 5.

### 3.1. Power Dependence at Low Temperature

Figure 1c shows typical spectra from the s-shell of a single QD taken at a low temperature (*T* = 3.2 K) and at a varied laser power *P*. We identify the peaks as follows: the first peak arising at low *P* and with the highest energy is related to an exciton (one electrostatically coupled electron–hole pair). The biexciton is composed of two excitons, it has less energy, and its intensity exceeds that of the exciton at higher *P*. The other peaks are related to charged excitons (trions) and multiexcitonic states caused by the beginning p-shell occupation. In Figure 1d, an example of a measured *P*-dependence of the X- and XX-peak intensities is plotted and Figure 1e shows the ratio of the peak intensities.

### 3.2. Temperature and Power Dependence

**Figure 2:**
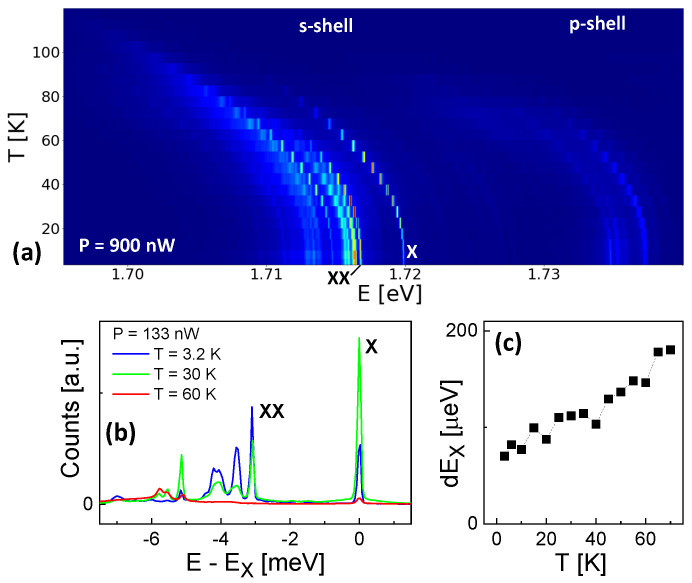
(**a**) Color-coded plot of a series of *T*-dependent spectra from a QD with $d_{F}$ = 0.33 nm. The exciton (X) and biexciton (XX) peaks are indicated. In addition to the X and XX lines, further multiexcitonic states in the s-shell and p-shell emission are visible due to the high excitation power of *P* = 900 nW. (**b**) Typical spectra from a QD with $d_{F}$ = 0.33 nm taken at *P* = 133 nW and varied temperature as indicated. The energy scale is normalized to the exciton energy $E_{x}$. (**c**) Lorentzian linewidth of the exciton peak at *P* = 133 nW as function of *T*.

Figure 2a shows a color-coded plot of the temperature-dependent emission of a QD with $d_{F}$ = 0.33 nm. The data were obtained at a high *P* = 900 nW to increase the intensity at elevated temperatures. Due to the strong excitation, in addition to the X and XX lines also further multiexcitonic states in the s-shell and even p-shell emission are visible. The X and XX peaks are determined by *P*-dependent measurements as is described in Section 3.1. The temperature-dependent shift of the emission energies agrees with the shift of the GaAs bandgap, which means that the QD quantization energies do not depend on *T*. Figure 2b shows typical spectra from the same QD but at a reduced *P* = 133 nW. As an important feature, the exciton intensity at *T* = 30 K is much higher in comparison to *T* = 3.2 K. This temperature enhancement is not observed for the biexciton intensity. Furthermore, the exciton peak shows only weak (see an example in Section 3.2) phonon sidebands [7,8,9,10,11] and an only slight broadening (Figure 2c) at elevated *T*. We note that the low-temperature linewidth of 70 µeV represents the resolution limit of our spectrometer. The influence of *T* and *P* on the X and XX intensities is discussed in more detail below.

**Figure 3:**
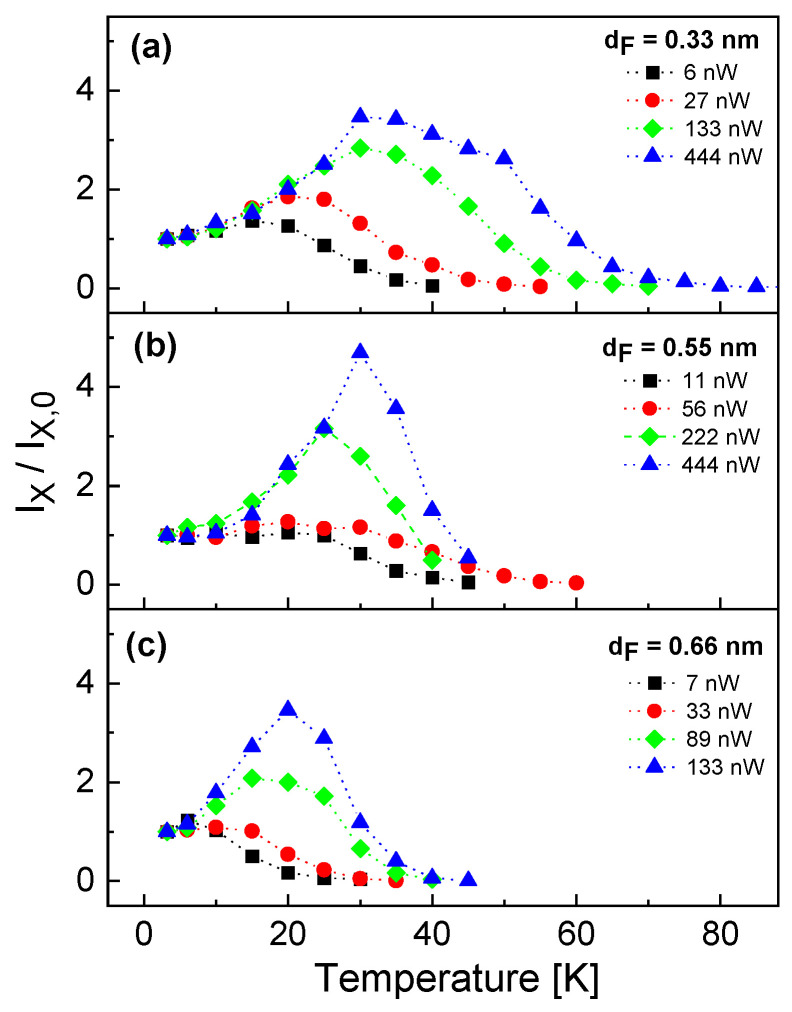
Temperature dependence of the exciton peak intensity $I_{x}$ normalized to the intensity $I_{x,0}$ at *T* = 3.2 K. The intensity represents the peak area of a Lorentzian fit after background subtraction. (**a**–**c**) Results from different QDs with varied filling layer thickness $d_{F}$ measured at the indicated excitation power *P*.

In Figure 3, the temperature dependence of the exciton intensity $I_{x}$ normalized to the intensity $I_{x,0}$ at *T* = 3.2 K is plotted for different QDs with varied filling layer thickness $d_{F}$ measured at varied excitation power *P*. For an increasing temperature, the data show that, first, $I_{x}/I_{x,0}$ increases up to a maximum intensity $I_{x,max}/I_{x,0}$ at a temperature $T_{x,max}$, which is followed by a strongly decreasing exciton intensity up to a complete disappearance of the signal. The unexpected increase in the intensity is explained below by an increasing excitation rate due to the onset of thermally driven diffusion of bulk excitons from the barrier layer into the dot. The decrease above $T_{x,max}$ is explained by loss channels like bulk-exciton break-off or thermal escape of charge carriers from the QD.

**Figure 4:**
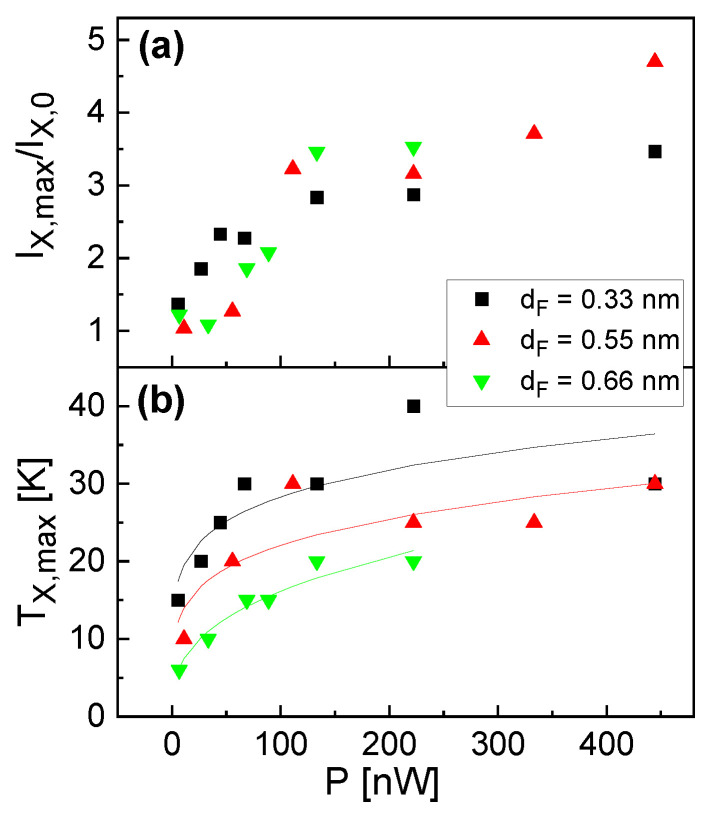
(**a**) Maximum exciton intensity $I_{x,max}/I_{x,0}$ taken from the *T*-dependent data in Figure 3; (**b**) temperature $T_{x,max}$ of the maximum exciton intensity (symbols) together with empirical fits in the form $aP^{b}$ (lines) for better visualization.

Furthermore, both $I_{x,max}/I_{x,0}$ and the corresponding $T_{x,max}$ increase with increasing excitation power. Figure 4 summarizes the maximum exciton intensity $I_{x,max}/I_{x,0}$ and the corresponding temperature $T_{x,max}$ as functions of the excitation power. We see again the increase in both quantities with increasing *P* up to a saturation at about *P* = 150 nW. Regarding the QD size, there is no clear dependence of $I_{x,max}/I_{x,0}$ on $d_{F}$, whereas $T_{x,max}$ shows larger values for a smaller $d_{F}$.

**Figure 5:**
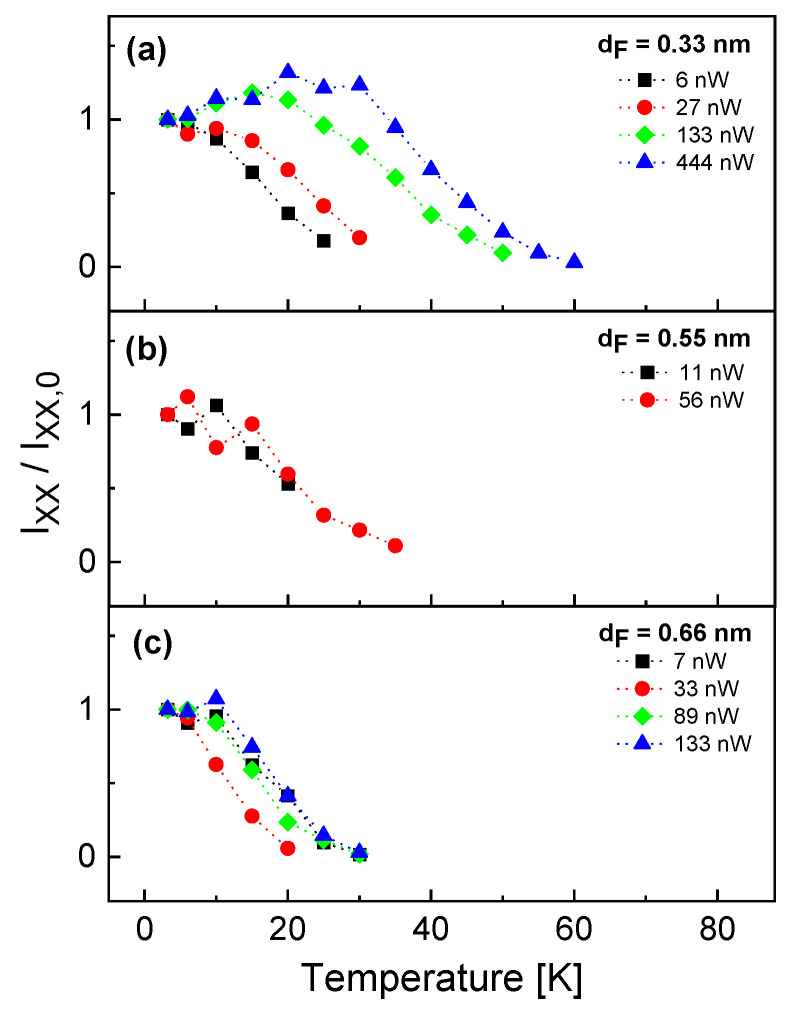
Temperature dependence of the biexciton peak intensity $I_{xx}$ normalized to the intensity $I_{xx,0}$ at *T* = 3.2 K. (**a**–**c**) Results from different QDs with varied filling layer thickness $d_{F}$ measured at the indicated excitation power *P*.

**Figure 6:**
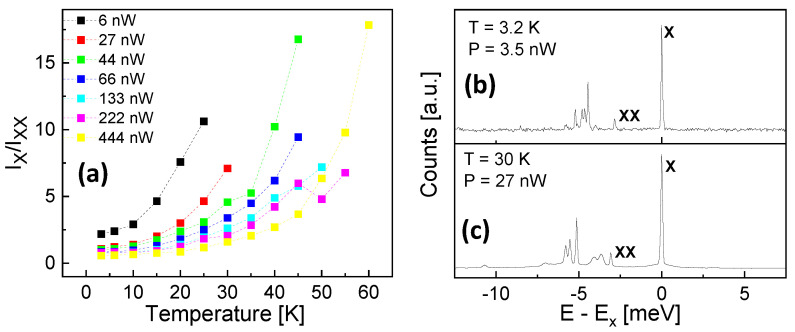
(**a**) Ratio of the measured exciton and biexciton intensities for a QD with $d_{F}$ = 0.33 nm as function of *T* at varied *P* as indicated. (**b**) PL spectrum of a QD with $d_{F}$ = 0.33 nm at *T* = 3.2 K and *P* = 3 nW. (**c**) PL spectrum of a QD with $d_{F}$ = 0.33 nm at *T* = 30 K and *P* = 27 nW. The energy scale is normalized to the exciton energy $E_{x}$.

In contrast with the exciton, the biexciton intensity shows, for a high excitation power, a slight increase with increasing *T*. At low *P*, the intensity decreases almost directly (Figure 5). For a further analysis of this effect, Figure 6a shows the ratio of the measured exciton and biexciton intensities as function of *T* at varied *P*. A strong increase of $I_{x}/I_{xx}$ with increasing *T* and a reduction with increasing *P* is clearly visible.

Figure 6b,c compare two scenarios to achieve a dominant exciton emission with $I_{x}/I_{xx}>$ 5. The exciton and biexciton peaks are identified by their *P*-dependence, as described in Section 3.1. The usual approach using a low temperature *T* = 3.2 K and a low excitation power *P* = 3.5 nW yields $I_{x}/I_{xx}$ = 6.6 and an exciton peak linewidth of $dE_{x}$ = 71 µeV. A measurement at a higher *T* = 30 K and *P* = 27 nW yields $I_{x}/I_{xx}$ = 7.1 and $dE_{x}$ = 109 µeV. So, the measurement a higher *T* maintains the peak ratio $I_{x}/I_{xx}$ but broadens the linewidth by about 50%. However, we note that the accuracy of the linewidth is rather low due to the resolution limit of the spectrometer. Other artifacts like phonon sidebands are very weak.

## 4. Model

The experimental data are interpreted on the basis of a rate model. The starting points are the exciton and biexciton peak intensities, which are described by the rate of radiative recombinations in a QD. Other considered processes are the generation of excitons via laser illumination, either directly inside the QD or indirectly via diffusion from the barrier material into the dot, as well as nonradiative loss channels via bulk exciton break-off and thermal escape of excitons from a dot.

### 4.1. QD Peak Intensities

The QD exciton (X) and biexciton (XX) peak intensities in terms of photons per time are given by the respective rates of radiative recombinations in the QD

(1) $$\begin{array}{ccc} I_{x} & = & c_{I}R_{x}n_{x}\\ I_{xx} & = & c_{I}R_{xx}n_{xx} \end{array}$$

with a constant $c_{I}$, the rates $R_{x}=1/\tau_{x},R_{xx}=1/\tau_{xx}$ of exciton and biexciton radiative recombinations, the exciton and biexciton radiative lifetimes $\tau_{x},\tau_{xx}$, and the populations $n_{x}$, $n_{xx}$ of the exciton and biexciton states, respectively. The time-dependent populations are given by

(2) $$\begin{array}{ccc} \frac{dn_{x}}{dt} & = & \left(1-n_{x}-n_{xx}\right)G-Gn_{x}-R_{x}n_{x}+R_{xx}n_{xx}-An_{x}\\ \frac{dn_{xx}}{dt} & = & Gn_{x}-R_{xx}n_{xx}-An_{xx} \end{array}$$

with the rate *G* of exciton generation in the QD and the rate *A* of exciton or biexciton nonradiative annihilation processes in the QD. Here, $(1-n_{x}-n_{xx})G$ characterizes the generation of excitons in non-occupied dot states, $Gn_{x}$ the exciton to biexciton transformation, $R_{x}n_{x}$, $R_{xx}n_{xx}$ the excition and biexciton radiative decay, and $An_{x}$, $An_{xx}$ exciton and biexciton nonradiative annihilation processes. In equilibrium (t→∞, $dn_{x}/dt=dn_{xx}/dt=0$), we get

(3) $$\begin{array}{ccc} n_{x} & = & \frac{(R_{xx}+A)G}{A^{2}+A\left(2G+R_{x}+R_{xx}\right)+G^{2}+GR_{xx}+R_{x}R_{xx}}\\ n_{xx} & = & \frac{G}{R_{xx}+A}n_{x} \end{array}$$

Here, the exciton generation rate *G* and the annihilation rate *A* are assumed to depend on temperature, whereas the radiative recombination rates $R_{X}$ and $R_{XX}$ are approximated as temperature-independent. The temperature dependence of *G* is related to bulk excitons created in the barrier material and governed by their diffusion and break-off. Exciton annihilation is described as thermally activated processes. The modeling of the temperature dependent rates is described in the following sections.

### 4.2. Exciton Generation

As the central approach to explain the temperature enhancement of the QD exciton emission, we assume that a QD collects excitons through direct generation inside the dot, plus additional bulk excitons that diffuse from the barrier material into the dot:

(4) $$G=G_{q}+G_{d},$$

where $G_{q}$ is the rate of direct exciton generation inside a QD and $G_{d}$ is the rate of exciton diffusion from the barrier material into a dot.

The laser illumination with power *P* means a flux of $n_{ph,0}=c_{p}P$ photons per unit of time and volume element towards the sample surface, with a constant $c_{P}$. The photon flux is attenuated inside the AlGaAs barrier material, which is described according to the Lambert–Beer law $n_{ph}\left(x\right)=n_{ph,0}\exp\left(-\alpha x\right)$, with the depth *x* below the surface, $n_{ph,0}=n_{ph}\left(x=0\right)=c_{p}P$, and the absorption coefficient α = 55,930 cm${}^{-1}$ (AlGaAs with an Al content of 0.315 at a laser wavelength of 532 nm [24]). In an undoped semiconductor, light is mainly absorbed by the formation of excitons. This gives the depth profile of the exciton generation rate $Q\left(x\right)=-dn_{ph}\left(x\right)/dx=\alpha \exp\left(-\alpha x\right)c_{P}P$. Assuming a spherical QD with a radius $r_{0}$ = 5 nm which is located *d* = 80 nm below the surface, the rate of direct exciton generation in a QD becomes

(5) $$G_{q}=\left(4/3\right)\pi r_{0}^{3}Q_{0}=\left(4/3\right)\pi r_{0}^{3}c_{\alpha }c_{P}P,$$

with $Q_{0}=Q\left(d+r_{0}\right)=c_{\alpha }c_{P}P$ and $c_{\alpha }=\alpha \exp\left(-\alpha \left[d+r_{0}\right]\right)$ = 0.00348 nm.

To evaluate the diffusion of excitons from the barrier material into a dot, we apply the approximation that the direct exciton generation rate inside a QD and in the surrounding barrier material is constant and equals $Q_{0}$. Far away from the dot, the density of bulk excitons in the barrier material is $n_{b,0}/dt=Q_{0}-n_{b,0}R_{b}-n_{b,0}A_{b}$, with the rate $R_{b}$ of bulk exciton radiative recombinations and the rate $A_{b}$, at which bulk excitons are annihilated by thermal break-off. In equilibrium (t→∞, $n_{b,0}/dt=0$), we get $n_{b,0}=Q_{0}/\left(R_{b}+A_{b}\right)$. For the bulk exciton radiative decay in AlGaAs, we assume a lifetime! $\tau_{b}=1/R_{b}=$ 1 ns [25] and for the annihilation of bulk excitons due to thermal break-off, we assume a rate $A_{b}=\nu_{A,b}\exp\left[-E_{A,b}/\left(k_{B}T\right)\right]$, where $\nu_{A,b}$ is a prefactor, $E_{A,b}$ is an activation energy, and $k_{B}$ is the Boltzmann’s constant. Now, we introduce the diffusion of excitons from the barrier material into a QD that is treated like a spherical sink with radius $r_{0}$. In steady-state the rate at which excitons diffuse towards the dot is $J=4\pi r^{2}Ddn_{b}/dr$, with the diffusion coefficient $D=\nu_{D}\exp\left[-E_{D}/\left(k_{B}T\right)\right]$, the prefactor $\nu_{D}$, the activation energy $E_{D}$, and the radial distance *r* to the QD center at r=0. This is solved by $n_{b}\left(r\right)=n_{b,0}-J/\left(4\pi rD\right)$. With the boundary condition $n_{b}\left(r_{0}\right)=0$ at the QD surface, we get $J=4\pi r_{0}Dn_{b,0}$. Now, the rate of indirect exciton generation in a QD by exciton diffusion from the barrier becomes

(6) $$G_{d}=4\pi r_{0}^{2}J={\left(4\pi \right)}^{2}r_{0}^{3}Dn_{b,0}={\left(4\pi \right)}^{2}r_{0}^{3}Dc_{\alpha }c_{P}P/\left(R_{b}+A_{b}\right).$$

### 4.3. Exciton Annihilation

Two mechanisms for the annihilation of generated excitons are considered. The thermal break-off in the barrier material was described above in Section 4.2. In addition, exciton annihilation can take place via thermal escape of charge carriers from a QD (see Equations (2) and (3)), which is already addressed in ref. [18]. The rate of thermal escape is described by $A=\nu_{A}\exp\left[-E_{A}/\left(k_{B}T\right)\right]$, where $\nu_{A}$ is a prefactor and $E_{A}$ an activation energy. Of course, there are other possible exciton loss-channels like Auger processes [26]. However, these are neglected here, since we do not expect a strong temperature dependence.

## 5. Model Results

This section addresses the application of the model for the interpretation of the experimental PL data.

### 5.1. Power Dependence at Low *T*

At low *T* = 3.2 K, several approximations can be applied to the model of Equation (3). In detail, the thermally activated loss channels *A* and the diffusion coefficient *D* for indirect exciton generation are negligibly small, which yields $G=G_{q}$. This simplifies Equation (3) to

(7) $$\begin{array}{ccc} n_{x,0} & = & \frac{G_{q}R_{xx}}{\left(R_{x}+G_{q}\right)R_{xx}+G_{q}^{2}}\\ n_{xx,0} & = & \frac{G_{q}}{R_{xx}}n_{x,0} \end{array}$$

Equations (1) and (7) allow the calculation of the X and XX power dependencies and a comparison with the experimental data in Figure 1b using four free model parameters $c_{I}$, $c_{P}$, $R_{x}$, and $R_{xx}$. To reduce the number of free parameters, we consider previous lifetime measurements [19] of similar QDs, which indicate $\tau_{x}=1/R_{x}$ = 0.39 ns for $d_{F}$ = 0.33 nm. The remaining parameters are determined by fitting: $\tau_{xx}=1/R_{xx}$ = 0.068 ns, $c_{P}$ = 0.0265 ns${}^{-1}$nW${}^{-1}$nm${}^{-2}$, and $c_{I}$ = 226.

Figure 1d shows a comparison of the model results with the experimental data at low *T* = 3.2 K. Despite its simplicity, the model demonstrates a good reproduction of the power dependence of $I_{x}$ and $I_{xx}$. Also, the power dependence of the intensity ratio $I_{x}/I_{xx}$ is well reproduced by the model (Figure 1e), where Equation (7) predicts $I_{x}/I_{xx}=R_{x}/G$. However, we note that the fitted value of $\tau_{xx}$ is unrealistic small. In a simple approximation, it can be assumed that $\tau_{xx}=\tau_{x}/2$ = 0.145 ns, since a biexciton with a doubled number of charge carriers has a doubled recombination rate in comparison to a single exciton. The inaccuracy of the fitted $\tau_{xx}$ can be related to the influence of states in higher shells that are not considered in the model.

### 5.2. Temperature and Power Dependence

The temperature dependence of the normalized exciton intensity in Figure 3 is modeled by $I_{x}/I_{x,0}$, where $I_{x}=c_{I}R_{x}n_{x}$ (Equation (3)) considers the temperature dependence and $I_{x,0}=c_{I}R_{x}n_{x,0}$ (Equation (7)) the intensity at low temperature *T* = 3.2 K, as is addressed above in Section 5.1. We use the model parameters $c_{I}$ = 226, $c_{P}$ = 0.0265 ns${}^{-1}$nW${}^{-1}$nm${}^{-2}$, as well as $\tau_{x}$ = 0.39 ns and $\tau_{xx}=\tau_{x}/2$ = 0.145 ns according to Section 5.1. Due to the number of remaining model parameters, the fitting is carried out in several steps.

**Figure 7:**
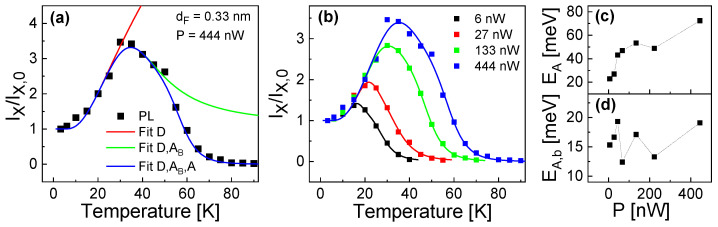
Normalized exciton intensity $I_{x}/I_{x,0}$ as function of *T*. (**a**) Comparison of experimental (PL) values with model results, where the processes considered in the model are varied. All model calculations include exciton generation in the QD ($Q_{q}$) and radiative recombinations $R_{x}$, $R_{xx}$. Fit *D* also considers exciton diffusion from the barrier into the QD, Fit $D,A_{b}$ considers bulk exciton break-off, and Fit $D,A_{b},A$ considers thermal escape of charge carriers from a dot. (**b**) Comparison of experimental (symbols) and calculated (lines) values at varied laser power *P* as indicated. (**c**,**d**) Fitted exciton-annihilation related activation energies as function of *P*.

In a first step, the bulk exciton diffusion-related parameters $\nu_{D}$ = 3.2 $\times \;10^{8}$ s${}^{-1}$ and $E_{D}$ = 4.2 meV are determined for best agreement with the approximately exponential growth of $I_{x}/I_{x,0}$ in the temperature range of *T* = 3.2...30 K. In a next step, the bulk exciton break-off-related parameters $\nu_{A,b}$ = 2.22 $\times \;10^{11}$ s${}^{-1}$ and $E_{A,b}$ = 20.5 meV are fitted in the temperature range of *T* = 3.2...50 K. And finally, the parameters $\nu_{A}$ = 3.22 $\times \;10^{15}$ s${}^{-1}$ and $E_{A}$ = 72 meV describing the thermal escape are fitted over the whole temperature range. Figure 7a visualizes the respective regimes at which the different processes have an influence and demonstrates the very good reproduction of the experimental temperature-dependence by the model.

However, as a significant difference from the experimental behavior (Figure 3), the model shows almost no influence of the excitation power *P* on the normalized exciton intensity $I_{x}/I_{x,0}$. Mathematically, this is caused by the normalization, where two quantities with similar *P*-dependence are divided. Obviously, the mechanism for the experimental *P*-dependence is not included in the model. Since the slope of the bulk exciton diffusion related increase does not depend on *P* (Figure 3a), we assume an additional *P*-dependence of either the bulk exciton break-off or the thermal escape. To test this, we have fitted the exciton-annihilation related parameters for other values of *P*. The resulting activation energies $E_{A,b}$ for bulk exciton break-off and $E_{A}$ for thermal escape are plotted in Figure 7c. In the figure, the values of $E_{A}$ show a clear increase with *P*, whereas $E_{A,b}$ is almost constant. We assume a mechanism where exciton annihilation by thermal escape is caused by a combination of several thermally activated processes, with the respective strengths being controlled by the excitation power.

## 6. Discussion and Conclusions

The temperature dependence of the exciton and biexciton peak intensities from GaAs cone–shell QDs is studied at varied excitation power and dot size. An interesting finding is a strong temperature enhancement of the exciton emission up to a factor of five. The maximum intensity and the corresponding temperature $T_{x,max}$ depend on excitation power and dot size. The experimental data are modeled by considering the competing processes of exciton generation, annihilation, and recombination. Exciton generation in the QDs takes place through direct excitation in the dot layer plus additional bulk excitons that diffuse from the barrier layers into the dot. The temperature-dependent bulk-exciton diffusion is the reason for the temperature-enhanced exciton emission. Above $T_{x,max}$, the intensity decreases due to exciton annihilation processes. In comparison to the exciton, the biexciton intensity shows an only very weak enhancement, which is attributed to a more efficient annihilation.

A temperature enhancement was also reported for the optical emission from GaAs quantum wells embedded in an AlGaAs barrier [25]. The enhancement was related to the same mechanism as depicted in the present study, i.e., generation of excitons in the barrier material and their temperature-driven diffusion into the quantum wells. Considering this mechanism, a temperature enhancement of the exciton intensity under nonresonant excitation is expected for all types of QDs which are embedded in a thick semiconductor barrier. Such epitaxial QDs are usually fabricated via MBE. Nevertheless, the literature which provides *T*-dependent PL data on various types of single epitaxial QDs [7,8,13,15,16] reports no temperature enhancement. Only in ref. [17] is a slight temperature enhancement observed for InAs/GaAs multilayer QDs. However, these measurements are taken using ensemble PL, which integrates the whole s-shell intensity, including charged excitons and multiexcitonic lines, and allows no clear relation to the pure exciton intensity. According to Figure 4, a probable reason for the absence of a temperature enhancement is a very low excitation power *P*, which is often used in the literature for studies focusing on the exciton behavior.

From a practical point of view, the enhancement of the exciton intensity can substantially simplify the spectroscopy of single QDs. A $T_{x,max}$ around 30 K allows us to substitute the usual expensive, large, and maintenance-intensive sample-cooling technologies (liquid helium or closed-cycle Gifford–McMahon cryostats) which is required for the spectroscopy at liquid helium temperature. With a base temperature around 30 K, spectroscopy of excitonic features has been made possible by inexpensive and compact Stirling cryocoolers with a long Mean Time To Failure (MTTF). The usage of Stirling cryocoolers also simplifies the development of devices for quantum information technology, where single QDs are utilized as a single-photon source [15]. On the other side, studies including the behavior of biexcitons are suggested at liquid-helium temperature, since the biexciton intensity shows an only very weak temperature enhancement and significantly degrades at elevated temperatures. Furthermore, it should be noted that in quantum information technology, the QDs are often studied using sub-barrier excitation, either resonant or quasi-resonant. In that case, the proposed mechanism of exciton enhancement via bulk exciton diffusion is not applicable.

## Figures and Tables

**Table 1:** The QD size-related parameters (Figure 1b) are calculated based on the deposited filling layer thickness $d_{F}$ according to ref. [23].

$d_{F}$	$h_{\mathrm{QD}}$	$d_{\mathrm{QD}}$	$r_{\mathrm{QD}}$
0.33 nm	9.3 nm	13.6 nm	24.5 nm
0.55 nm	11.6 nm	17.2 nm	32.0 nm
0.66 nm	12.8 nm	19.0 nm	35.0 nm

## Data Availability

The data presented in this study are available on request from the corresponding author.
